# The association between HIV infection and precancerous cervical lesion. A systematic review and meta‐analysis of case–control studies

**DOI:** 10.1002/hsr2.1485

**Published:** 2023-08-02

**Authors:** Habtamu Geremew, Hiwot Tesfa, Misganaw A. Mengstie, Chalachew Gashu, Yoseph Kassa, Abraham Negash, Anteneh Mengist Dessie, Demeke Geremew

**Affiliations:** ^1^ College of Health Science Oda Bultum University Chiro Ethiopia; ^2^ Department of Public Health, College of Medicine and Health Sciences Injibara University Injibara Ethiopia; ^3^ Department of Biochemistry, College of Medicine and Health Sciences Debre Tabor University Debre Tabor Ethiopia; ^4^ Department of Statistics, College of Natural and Computational Science Oda Bultum University Chiro Ethiopia; ^5^ School of Nursing and Midwifery, College of Health and Medical Sciences Haramaya University Harar Ethiopia; ^6^ Department of Public Health, College of Health Sciences Debre Tabor University Debre Tabor Ethiopia; ^7^ Department of Medical Laboratory Science, Immunology and Molecular Biology Unit, College of Medicine and Health Sciences Bahir Dar University Bahir Dar Ethiopia

**Keywords:** Ethiopia, HIV infection, Meta‐analysis, Precancerous cervical lesion

## Abstract

**Background:**

The effect of human immunodeficiency virus (HIV) on precancerous cervical lesion is not consistent across studies. Besides to the variability in the presence of a significant association between HIV and precancerous cervical lesion, the reported strengths are inconsistent among studies that report a significant association. Therefore, we sought to determine the impact of HIV on women's risk of precancerous cervical lesion by conducting a systematic review and meta‐analysis of case–control studies in Ethiopia.

**Methods:**

Relevant articles were systematically searched on African Journals Online, Cochrane Library, Science Direct, Google Scholar, and PubMed from January 1, 2023, to February 20, 2023. After critical appraisal, pertinent data were extracted into an Excel spreadsheet and then exported to STATA 14 for further statistical analysis. The pooled effect size was estimated using the random‐effect model. The Egger's regression test and *I* ^2^ statistics were employed to assess publication bias and heterogeneity among included studies, respectively.

**Results:**

Ten case–control studies with a total of 3035 participants (992 cases and 2043 controls) were involved in this meta‐analysis. According to our analysis, HIV‐infected women were 2.86 times more likely to develop precancerous cervical lesion as compared with their counterparts (odds ratio: 2.86, 95% confidence interval: 1.79, 4.58).

**Conclusion:**

We found that HIV‐infected women have a higher risk of precancerous cervical lesion. Thus, targeted screening programs should be considered to reduce the burden of cervical cancer among HIV‐infected women in Ethiopia.

## 1 INTRODUCTION

Cervical cancer is a malignant tumor that starts in the cells of the cervix.^1^ It is a major global public health threat, causing about 342,000 deaths and 604,000 reported cases of the disease in 2020 alone.^2^ Cervical cancer continued to be the common cause of maternal morbidity and mortality in underdeveloped settings, including sub‐Saharan Africa.^3^, ^4^ In Ethiopia, cervical cancer was the second‐most frequent cancer in 2020.^2^

Early detection and management of the precancerous cervical lesion is an effective way of preventing this life‐threatening disease.^3^ Women who present to care with an advanced disease stage have a higher risk of mortality and a poor prognosis.^5^, ^6^ However, more than half of women in Ethiopia present for care at a later stage of the disease.^7^

Given the low uptake of human papillomavirus (HPV) vaccination among eligible women in Ethiopia, early determination of the precancerous lesion forms the foundation for the prevention and control of this important disease.^8^, ^9^ Besides, the utilization of cervical cancer screening service is very low in Ethiopia.^10^ Hence, investigating the determinants of precancerous cervical lesion is useful to tailor more focused screening programs.^11^

Human immunodeficiency virus (HIV) infection is one of the important determinants of precancerous lesions of the cervix.^12^, ^13^ However, the findings are not consistent throughout the studies. For instance, studies conducted in Ruanda and Ethiopia revealed that HIV‐infected women have a higher risk of precancerous cervical lesion.^14^, ^15^ Conversely, these reports are argued by the findings of other researchers.^16^, ^17^

Besides to the variability in the presence of a significant association between HIV infection and precancerous cervical lesion, the strengths of the reported measures of association are also different across studies that report significant associations. Thus, showing inconclusive evidence about the effect of HIV infection on the risk of precancerous cervical lesion. Therefore, this study aimed to estimate the pooled effect size of HIV infection on women's risk of precancerous cervical lesion by conducting a systematic review and meta‐analysis of case–control studies in Ethiopia.

## 2 METHODS AND MATERIAL

### 2.1 Protocol registration and reporting

The preferred reporting item for systematic review and meta‐analysis was closely followed to report this review.^18^ The protocol for this systematic review and meta‐analysis was registered in the International Prospective Register of Systematic Reviews (PROSPERO) database with protocol number, CRD42023397345.

### 2.2 Search strategy

A comprehensive, systematic electronic search was made from January 1, 2023, to February 20, 2023, on the following databases: African Journals Online, Cochrane Library, Science Direct, Google Scholar, and PubMed to retrieve relevant articles. The keywords used during the search include: “Determinant,” “Predictor,” “Risk factor,” “associated factor,” “Precancerous cervical lesion,” “Uterine cervical dysplasia,” “Cervical lesion,” “Cervical cancer,” “VIA Positive,” “Pap smear positive,” and “Ethiopia.” The Boolean operators were utilized to develop search strings. Besides, reference lists of important articles were also scrutinized to identify additional studies.

### 2.3 Eligibility criteria

All case–control studies that investigate the association between HIV infection and precancerous cervical lesion in the Ethiopian setting were included in this review. All articles published in the English language were considered in this study without restriction to their study period. However, cross‐sectional studies, systematic reviews, duplicate studies, editorials, and studies conducted only among HIV‐positive women were not included in this systematic review and meta‐analysis.

### 2.4 Variables

Our exposure of interest was the HIV status of women and our outcome of interest was precancerous cervical lesion. Visual inspection of the cervix with acetic acid (VIA) was used to ascertain the outcome. Hence, cases were women who had positive VIA screening test results and controls were women who were negative for the VIA screening test.^19^

### 2.5 Study selection, quality appraisal, and data extraction

After removing duplicates, articles were initially reviewed by their title and abstract. For articles that were deemed suitable by title and abstract, the full texts were assessed to identify potential articles for inclusion in this analysis. Endnote version X7.2 was used to combine database search results, remove duplicate studies, and manage the overall citation process.

The Joanna Brigg's Institute (JBI) quality assessment checklist for case–control studies was employed to verify the quality of the included studies.^20^ Two review team members (HG and HT) assessed the quality of the included studies, and a third author (MAM) was involved whenever disagreements arose between the two authors.

A standardized data extraction form that was developed by considering the JBI guide for data extraction and synthesis was used for the abstraction of the necessary information from primary studies.^21^ Data from original studies were retrieved by two independent reviewers into an Excel spreadsheet and included the name of the primary author, year of publication, region, data collection method, sample size, number of cases, number of controls, and statistical significance of the association between HIV and precancerous cervical lesion. Moreover, data regarding the exposure status of cases and controls were also retrieved in the form of two by two tables.

### 2.6 Statistical analysis

After extraction, the data were exported to STATA 14 software for further statistical analysis. The random effect model (DerSimonian‐Laird method) was used to estimate the pooled effect size so as to account for the observed heterogeneity among the included studies.^22^ Heterogeneity between included studies was assessed using the *I* ^2^ statistics, and it was regarded as high, moderate, or low when the *I* ^2^ test statistics results were 75%, 50%, or 25%, respectively.^23^ Funnel plot and Egger's regression test were used to assess the presence of publication bias.^24^ Furthermore, the influence of each study on the overall meta‐analysis estimate was assessed using sensitivity analysis.

## 3 RESULTS

### 3.1 Search result

**Figure 1:**
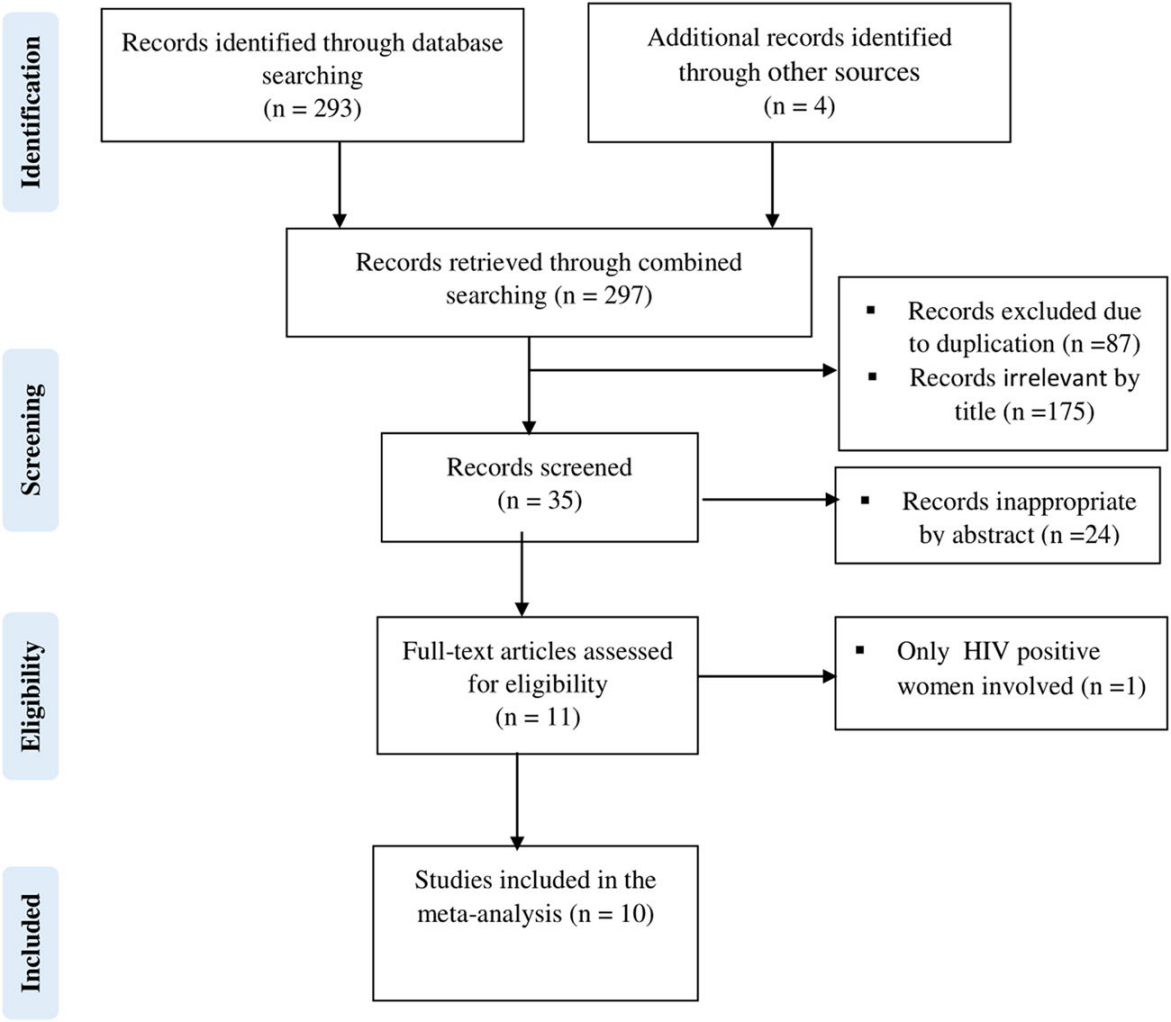
PRISMA flow chart for the studies identified, screened, and included.

Our electronic search produced a total of 297 articles. Of these, 87 studies were eliminated due to redundancy. Screening of 210 articles by their title and abstract resulted in the removal of 199 studies. Then, the full texts of the remaining 11 studies were reviewed against the preset inclusion criteria and 1 study was removed, because it involved only HIV‐infected women.^25^ Finally, 10 studies were included in this meta‐analysis (Figure 1).

### 3.2 Characteristics of original articles

This review included 10 case–control studies with a total of 3035 participants (992 cases and 2043 controls). All of the included studies employed VIA to verify the presence of precancerous cervical lesion. Two studies used record review (secondary data) to collect the necessary information.^17^, ^26^ Regarding the study area: four studies were from the Amhara region,^19^, ^27^, ^28^, ^29^ three studies were from the Oromia region,^16^, ^17^, ^30^ two studies were from the Southern Nations Nationalities and Peoples region,^26^, ^31^ and one study was from Addis Ababa.^11^ Moreover, only three studies reported a significant association between HIV infection and precancerous cervical lesion; yet the adjusted odds ratio ranges from 3.41 to 7.41^26^, ^27^, ^30^ (Table 1).

**Table 1:** Characteristics of studies included in the meta‐analysis (*n* = 10).

Primary author	Publication year	Region	Data collection method	Sample size	Number of cases	Number of controls	Association b/n HIV & PCL	Quality assessment
Alamiraw et al.^29^	2020	Amhara	Interview	407	102	305	Not significant	High
Beyene et al.^31^	2021	SNNP	Interview	295	98	197	Not significant	High
Dejene et al.^26^	2020	SNNP	Record review	520	260	260	Significant	High
Kassa et al.^17^	2018	Oromia	Record review	164	55	109	Not significant	High
Mengistu et al.^28^	2022	Amhara	Interview	410	82	328	Not significant	High
Taye et al.^19^	2021	Amhara	Interview	200	67	133	Not significant	High
Teame et al.^11^	2018	Addis Ababa	Interview	343	114	229	Not significant	High
Tekalegn et al.^16^	2020	Oromia	Interview	222	74	148	Not significant	High
Teklehaimanot et al.^27^	2022	Amhara	Interview	216	54	162	Significant	High
Tesfaye et al.^30^	2022	Oromia	Interview	258	86	172	Significant	High

Abbreviations: HIV, human immunodeficiency virus; PCL, precancerous cervical lesion; SNNP, Southern Nations Nationalities and Peoples.

### 3.3 Association between HIV infection and precancerous cervical lesion

**Figure 2:**
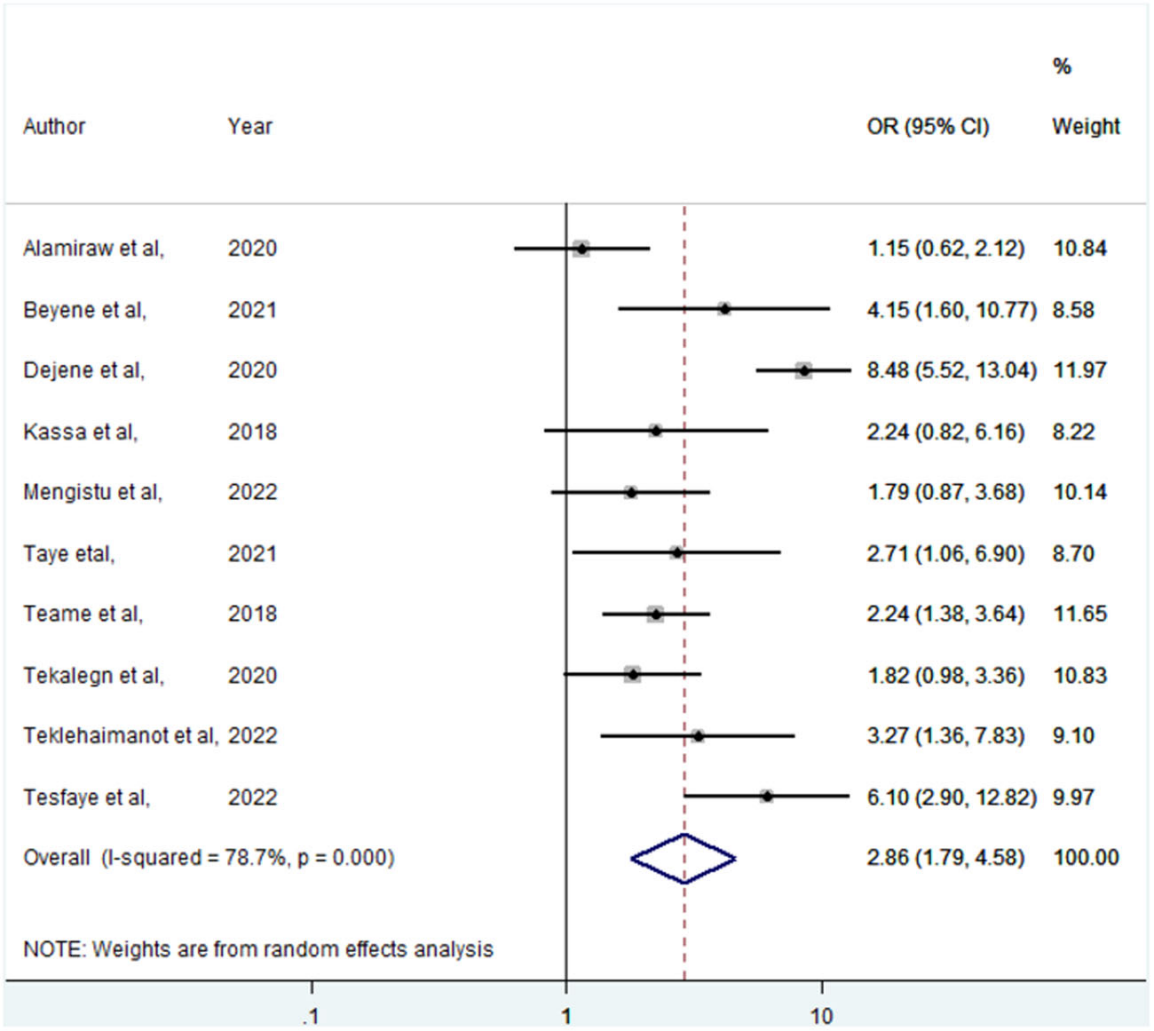
Forest plot for the pooled effect size of human immunodeficiency virus infection on precancerous cervical lesion. CI, confidence interval; OR, odds ratio.

Using 10 studies conducted in Ethiopia, this meta‐analysis found that there is a significant association between HIV infection and precancerous cervical lesions. The included studies exhibited considerable heterogeneity (*I* ^2^ = 78.7%, *p* < 0.001). Thus, the random‐effect meta‐analysis model was used to compute the pooled odds ratio. Accordingly, HIV‐infected women were about three (odds ratio = 2.86, 95% confidence interval: 1.79, 4.58) times more likely to develop precancerous cervical lesions than HIV‐negative women (Figure 2).

### 3.4 Publication bias and sensitivity analysis

**Figure 3:**
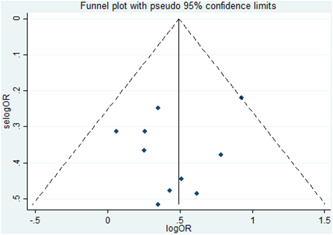
Funnel plot, assessing the existence of publication bias.

In this analysis, the funnel plot and Egger's regression test were used to assess the presence of publication bias. Consequently, the results indicate no publication bias, as demonstrated by the symmetrical funnel plot (Figure 3) and insignificant Eggers regression test (*p* = 0.43).

**Figure 4:**
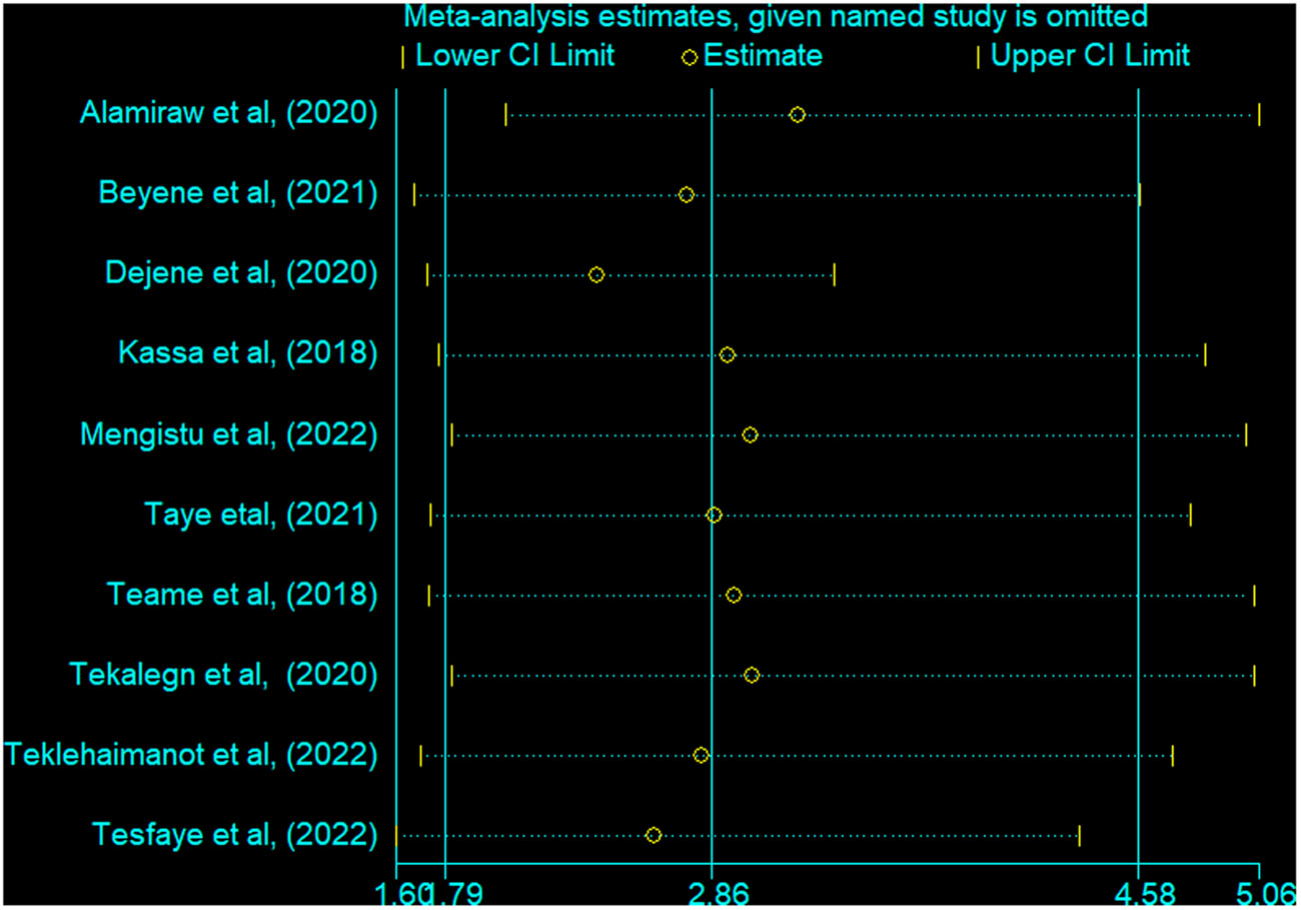
Sensitivity analysis for the impact of human immunodeficiency virus on precancerous cervical lesion. CI, confidence interval.

The effect of each study on the pooled effect size estimate was investigated using sensitivity analysis. As a result, the pooled effect size estimate of HIV infection on precancerous cervical lesion was steady and reliable when analyzed by removing one study at a time (Figure 4).

## 4 DISCUSSION

Ethiopia is one of the countries with the highest burden of HIV infection.^32^ Cervical cancer is also a major health threat in the country.^33^ HIV‐infected women are substantially affected by cervical cancer and precancerous lesions of the cervix, posing a synergetic impact on their well‐being.^34^ Therefore, investigating the association between HIV infection and precancerous cervical lesion is important to design more focused care for the prevention and control of this disastrous disease. In this regard, our meta‐analysis assessed the impact of HIV infection on women's risk of precancerous cervical lesion using case–control studies in Ethiopia.

According to our analysis, HIV‐infected women had a higher risk of precancerous cervical lesion as compared with women who were not infected by HIV. This finding is supported by the results of another meta‐analysis.^13^ The possible explanations could be due to the unfavorable effect of HIV‐related immunosuppression on the natural history of HPV, which has a significant correlation with increased persistence and acquisition of HPV infection.^35^, ^36^ The shared behavioral risks of sexual transmission for both infections could also be another plausible elucidation.^37^ Moreover, HIV‐infected women are more prone to high‐risk HPV infections, hence exacerbating their risk of developing precancerous cervical lesion and/or cervical cancer.^38^ This justification is further strengthened by the findings of a meta‐analysis that revealed an elevated rate of high‐risk HPV infection among HIV‐infected women.^39^

Women infected with HIV also have a higher risk of adverse transition from infection with HPV to the cervical lesion.^40^ Analysis of multiple cohort studies conducted in Senegal showed that HIV infection is significantly associated with a more than twofold increase in the hazard of progression from HPV infection to the development of high‐grade squamous intraepithelial lesions of the cervix.^41^

Studies also revealed that HIV‐positive women with a low CD4 count and/or a higher viral load were associated with an increased risk of HPV acquisition and cervical carcinogenesis.^38^ This might be due to the increased likelihood of HPV reactivation among immunosuppressed individuals.^42^ Thus, suggesting the importance of timely and proper antiretroviral therapy to prevent the development of cervical cancer.^43^

Generally, HIV infection is associated with a higher risk of precancerous cervical lesion and faster progression to the malignant forms. Hence, customized approaches are needed to reduce the morbidity and mortality of HIV‐infected women due to cervical cancer.

### 4.1 Strength and limitations

All studies employed the same method to ensure the occurrence of the outcome, thereby demonstrating clinical homogeneity among the included studies. On the other hand, inclusion of articles published only in the English language might affect its representativeness. Besides, none of the included studies were exposure specific.

## 5 CONCLUSION

We found that HIV‐infected women have a higher risk of precancerous cervical lesion. Thus, strengthening targeted screening for precancerous cervical lesion and integrating the service with lifelong antiretroviral care should be considered to reduce the burden of cervical cancer among HIV‐infected women in Ethiopia.

## AUTHOR CONTRIBUTIONS

**Habtamu Geremew**: Conceptualization; data curation; formal analysis; investigation; methodology; project administration; resources; software; validation; visualization; writing—original draft; writing—review and editing. **Hiwot Tesfa**: Data curation; investigation; methodology; project administration; resources; writing—review and editing. **Misganaw A. Mengstie**: Data curation; investigation; methodology; project administration; resources; writing—review and editing. **Chalachew Gashu**: Data curation; methodology; project administration; resources; visualization; writing—review and editing. **Yoseph Kassa**: Investigation; methodology; project administration; resources; visualization; writing—review and editing. **Abraham Negash**: Data curation; investigation; methodology; project administration; validation; writing—original draft; writing—review and editing. **Anteneh Mengist Dessie**: Investigation; methodology; project administration; validation; writing—original draft; writing—review and editing. **Demeke Geremew**: Conceptualization; data curation; formal analysis; investigation; methodology; software; writing—original draft; writing—review and editing.

## CONFLICT OF INTEREST STATEMENT

The authors declare no conflict of interest.

## ETHICS STATEMENT

Not applicable.

## TRANSPARENCY STATEMENT

The lead author Habtamu Geremew affirms that this manuscript is an honest, accurate, and transparent account of the study being reported; that no important aspects of the study have been omitted; and that any discrepancies from the study as planned (and, if relevant, registered) have been explained.

## Data Availability

Any data related to this manuscript will be accessible by requesting the corresponding author.
